# Selenium isotope evidence for progressive oxidation of the Neoproterozoic biosphere

**DOI:** 10.1038/ncomms10157

**Published:** 2015-12-18

**Authors:** Philip A. E. Pogge von Strandmann, Eva E. Stüeken, Tim Elliott, Simon W. Poulton, Carol M. Dehler, Don E. Canfield, David C. Catling

**Affiliations:** 1 Institute of Earth and Planetary Sciences, University College London and Birkbeck, University of London, Gower Street, London WC1E 6BT, UK; 2 Bristol Isotope Group, School of Earth Sciences, Bristol University, Wills Memorial Building, Queen's Road, Bristol BS8 1RJ, UK; 3 Department of Earth and Space Sciences, University of Washington, Seattle, Washington 98195, USA; 4 School of Earth and Environment, University of Leeds, Leeds LS2 9JT, UK; 5 Department of Geology, Utah State University, Logan, Utah 84322, USA; 6 NordCEE, University of Southern Denmark, 5230 Odense M, Denmark

## Abstract

Neoproterozoic (1,000–542 Myr ago) Earth experienced profound environmental change, including ‘snowball' glaciations, oxygenation and the appearance of animals. However, an integrated understanding of these events remains elusive, partly because proxies that track subtle oceanic or atmospheric redox trends are lacking. Here we utilize selenium (Se) isotopes as a tracer of Earth redox conditions. We find temporal trends towards lower *δ*^82/76^Se values in shales before and after all Neoproterozoic glaciations, which we interpret as incomplete reduction of Se oxyanions. Trends suggest that deep-ocean Se oxyanion concentrations increased because of progressive atmospheric and deep-ocean oxidation. Immediately after the Marinoan glaciation, higher *δ*^82/76^Se values superpose the general decline. This may indicate less oxic conditions with lower availability of oxyanions or increased bioproductivity along continental margins that captured heavy seawater *δ*^82/76^Se into buried organics. Overall, increased ocean oxidation and atmospheric O_2_ extended over at least 100 million years, setting the stage for early animal evolution.

## Abstract (web summary)

Our understanding of the conditions that characterised Neoproterozoic Earth are hindered by a lack of suitable proxies. Here, the authors propose and demonstrate the use of selenium isotopes as tracers of Earth's redox conditions.

## Graphical abstract

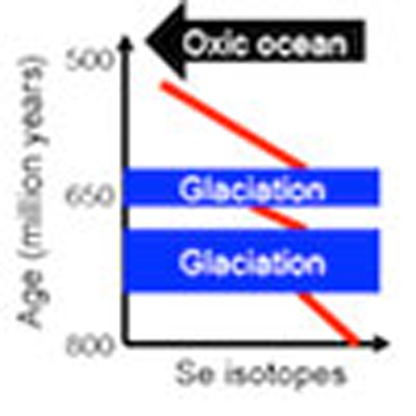

The history of atmospheric and oceanic redox is strongly interrelated with controls on biological evolution. One of the most significant changes in redox conditions occurred during the late Neoproterozoic Era^1^, alongside low-latitude ‘Snowball Earth' glaciations and the emergence of metazoans^2^. The surface ocean is generally considered to have been oxygenated long before the first Neoproterozoic glaciation, the Sturtian, occurring ∼716 Myr ago^1^,^3^,^4^. However, deeper waters remained anoxic and ferruginous (Fe^2+^ containing) for much of the Neoproterozoic, with fully oxic conditions becoming widespread (but not global) after the Gaskiers glaciation at ∼580 Myr ago^5^. Extensive deep-ocean oxygenation in the aftermath of the Gaskiers glaciation may have been a consequence of a rise in atmospheric oxygen driven by an influx of nutrients to the ocean during glacial melting^6^. Oxygenation may then have permitted the emergence of animal motility leading to predation and bioturbation, as well as biomineralization^1^. A possible rise in atmospheric oxygen at this time is supported by chromium isotope systematics during the later Neoproterozoic^7^,^8^, while an increased range in sulphur isotope compositions of biogenic pyrite has been interpreted to reflect increased atmospheric oxygen penetration into nearshore sediments^9^,^10^. Critically, however, proxies that can temporally resolve the progressive oxidation of the late Neoproterozoic and early Cambrian ocean are lacking, as existing proxies often require specific depositional conditions, or are only applicable to tracking more extreme redox changes such as identifying fully anoxic or oxic conditions.

Selenium (Se) isotopes in marine shales are a novel tracer of ocean and atmospheric oxygenation across the Neoproterozoic Oxygenation Event^11^,^12^,^13^,^14^. Selenium has four oxidation states: +6, +4, 0 and −2, and although the biogeochemical Se cycle shares similarities with that of sulphur, there are distinct differences, including redox transitions. Se oxyanion reduction (both Se^VI^ and Se^IV^) occurs at significantly higher Eh than the reduction of sulphate to sulphide^15^, and is closer to that of Fe^3+^ to Fe^2+^ (Fig. 1). The combination of multiple redox states, high redox potential and relatively low abundance may also make Se highly sensitive to redox changes^16^. This sensitivity and associated fractionation mechanisms mean that Se isotopes should have responded to the oxygenation of the deep ocean in the Neoproterozoic when the extent of euxinic and ferruginous water masses contracted significantly^5^.

In the modern surface ocean, Se exists as selenite (Se^IV^) or selenate (Se^VI^) anions (collectively denoted SeO_*x*_^ 2−^) and dissolved organic Se (denoted Se_org_). These species have different behaviour and fates depending on the oxygen levels in the water column. Selenium oxyanions have a short, nutrient-like behaviour and residence time (1,100–9,400 years, although a recent estimate suggests 26,000 years during the Phanerozoic)^15^,^17^,^18^. Organic selenium is common and generally dominates^15^,^19^ in surface waters, but is reoxidized at depth in oxic water columns (Fig. 2a) and has very low concentrations below the modern photic zone^20^. In modern anoxic water columns, in contrast, the remineralization of organic matter is limited, so that Se_org_ dominates below the chemocline^16^,^20^. The sedimentary archive consists of organic and reduced Se, where the former dominates in sediments deposited in anoxic environments^16^.

About 85% of the input flux of Se to the oceans (Fig. 2b) arises from rivers, through a combination of organic-bound Se^16^ and oxidative weathering of continental selenides to SeO_*x*_^ 2−^; almost all the rest comes from subaerial volcanism via rainfall. Hydrothermal fluid input is small (<1% (ref. 16)), and in any case its Se is probably scavenged by minerals close to the vent^21^,^22^. It has also been suggested that hydrothermal fluxes were insignificant in the Archaean and earlier Proterozoic^13^,^16^.

Here we reconstruct redox across the second oxygen rise, using marine shale Se isotope ratios. These are novel tracers of oceanic redox conditions, where lighter isotope ratios in shales are generally indicative of a large pool of oxyanions, implying more oxic conditions. There is an overall trend towards lighter isotope ratios from before the Sturtian glaciation to the Ediacaran–Cambrian boundary. Where time periods are covered by data from more than one sample suite, it suggests that the sediment Se isotopes reflect global redox changes. Therefore, we suggest that the global ocean became more oxic from at least the end of the Marinoan glaciation, and that, rather than a sudden redox change, the shift towards deep-ocean oxia was spread over a timescale of ∼100 Myr ago.

## Results

### Samples

We report Se isotope ratios and Se concentrations for marine shales from seven different geological sections that together span from 770 to 525 Myr ago. Sample suites include the Mackenzie Mountains, Canada (the Twitya and Sheepbed formations), the Yangtze Platform (comprising samples from the Doushantuo, Luichapo and Niutitang formations), Tapley Hill, Australia and finally from the Mineral Fork Formation, and Uinta Mountain and Chuar groups, western USA.

Both formations from Canada are a part of the Windermere Supergroup. The Twitya Formation represents continental slope deposition on a passive margin, where water depths may have reached ∼1 km (ref. 23). In contrast, the Sheepbed Formation was deposited in a deep-water outer shelf setting^23^. Both Formations contain assemblages of spheromorphic acritarchs, as well as Ediacara-type fossils. The Yangtze Platform, China, comprises several formations according to the nomenclature of ref. 5 (Niutitang, Doushantuo and Liuchanpo). They are thought to represent deposition on the lower continental slope to deep basin of a passive margin setting^24^,^25^. The Tapley Hill Formation is from the Adelaide Rift Complex, Australia. It was deposited in a relatively shallow epicontinental basin of about 200–300 km width, with apparent access to the global ocean^26^.

Four members of the Chuar Group (Grand Canyon, USA) were analysed, including samples from the Walcott Member (742 Myr ago^27^), the Awatubi Member (∼745 Myr ago upper, 750 Myr ago lower^28^), the Carbon Canyon Member (∼755 Myr ago) and the Jupiter Member (760 Myr ago^28^). The group was deposited in an intracratonic extensional basin between ∼740 and 780 Myr ago^29^. The depositional setting was marine to marginal marine, with access to the open ocean during transgressions, recorded by both sedimentological (wave and tide influenced deposition) and palaeontological evidence^30^.

Samples from three different Formations of the Uinta Mountain Group (Utah, USA) were also analysed. The group correlates with the Chuar Group, using sedimentological, geochemical, palaeontological and geochronological data^31^. The Uinta Mountain Group was deposited in an intracratonic extensional basin during the early stages of the breakup of Rodinia^31^. The quartzose shale of the Red Pine Shale (sample age 740 Myr ago, based on correlation with the Chuar Group) indicates a prodelta marine environment^31^. The sample from the Moosehorn Lake formation (sample age 765 Myr ago, based on correlation with eastern Uinta Mountain Group formation of Outlaw Trail^31^) represents a marine setting, which was connected to the open ocean. Finally, the Deadhorse formation (∼760 Myr ago, stratigraphically above the Moosehorn Lake Formation) represents a barrier bar environment, and as such can be considered to be marine, albeit nearshore^31^.

Finally, a single sample from the Mineral Fork formation was analysed. Zircons in this Formation have been dated at ∼700 Myr ago, and other detrital zircon peaks in correlative successions are dated at 700–680 Myr ago^32^. Hence, while using the earlier dates of the Sturtian onset from ref. 4 (based partly on Twitya) of ∼717 Myr ago, the maximum depositional age of the Mineral Fork (∼700 Myr ago) suggests a later glacial effect further south^32^.

Sample chronology is taken from several studies^5^,^31^,^32^. In general, these are based on correlation between absolute ages of individual dated layers. The sample dates between the Sturtian and Marinoan have been adjusted to the new timing of the Sturtian^4^, by maintaining the relative timing from ref. 5, and condensing the absolute ages.

### Shale Se isotope record

**Figure 3:**
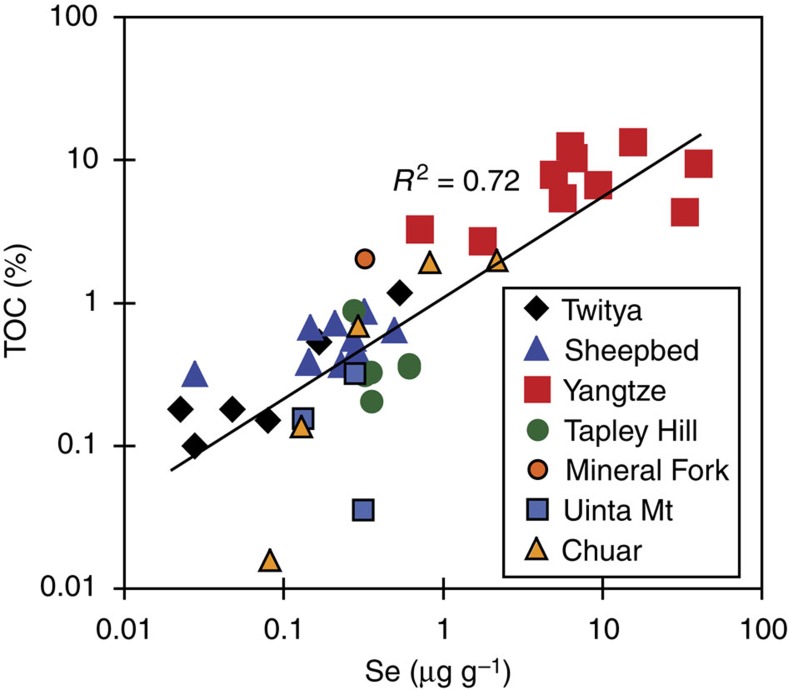
Correlation between total organic carbon and Se concentrations. The co-variation with TOC (total organic carbon), for all samples from all of this study's different sample locations, suggests that Se_org_ is an important Se phase in sediments.

**Figure 4:**
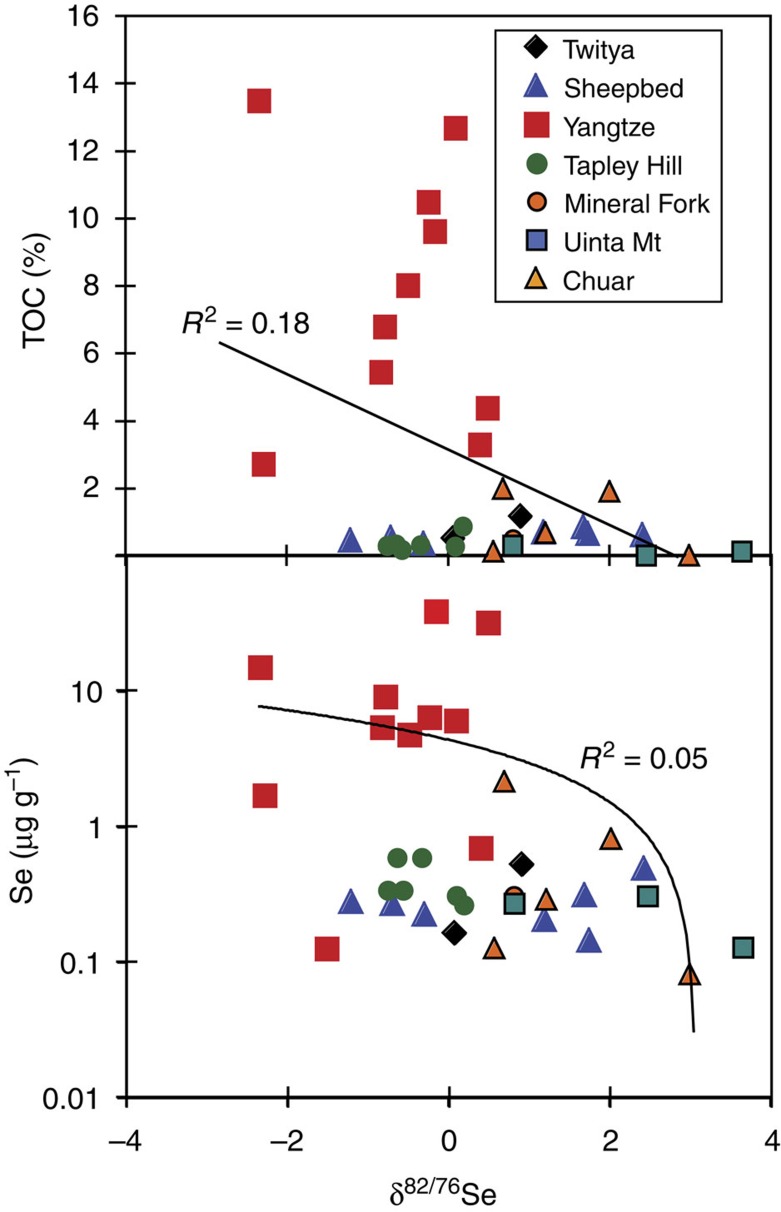
The lack of a linear relationship between Se isotopes and TOC and Se concentrations. The lack of correlations between any of the parameters, as demonstrated by the *R*^2^ values and trend lines, shows different controls on Se isotopes from those that control Se concentrations.

Selenium concentrations in our sample shales vary widely (0.02–39.5 μg g^−1^), with some correlation between [Se] and TOC (total organic carbon; Table 1; Fig. 3). This suggests that some sedimentary Se was buried bound to organic matter, as expected^12^,^33^. There is also a generally positive, but poor (*r*^2^=0.2) relationship between [Se] and the proportion of highly reactive Fe bound in pyrite, suggesting a weaker effect of substitution for S in pyrite. However, none of these parameters correlate with Se isotope ratios (Fig. 4), showing that *δ*^82/76^Se are controlled differently from Se concentrations, which again is anticipated for Se behaviour and may reflect changing reservoir effects over time or between basins^12^,^16^.

**Figure 5:**
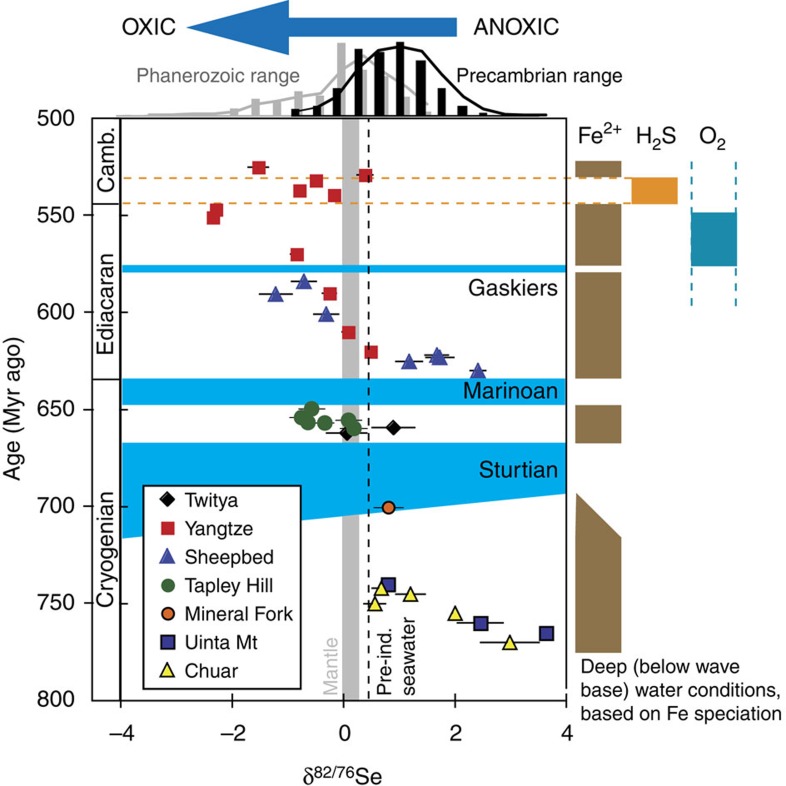
Se isotope data for Late Neoproterozoic marine sediments. The vertical grey bar represents the mantle *δ*^82/76^Se^43^, and the vertical dashed line represents likely pre-industrial modern seawater *δ*^82/76^Se^12^,^22^. Blue horizontal bars represent glacial intervals. The black histogram shows the range of Precambrian *δ*^82/76^Se^13^, while the grey histogram shows the range of Phanerozoic shale data^12^,^14^. Deep-water redox conditions are also shown on the right, based on Fe speciation data for the same sediments. Ferruginous deep-water conditions dominate around the Sturtian and Marinoan glaciations. Post-Gaskiers redox conditions are more variable globally, with evidence for widespread ocean oxygenation, but a continuation of ferruginous conditions in some basins. The diachronous initiation of the Sturtian glaciation is based on the geological evidence (see Sample section).

The measured range of our Neoproterozoic samples (−2.35 to +3.63‰, where the Se isotope ratio is reported relative to NIST-3149 (refs 34, 35, 36); Fig. 5; Table 1) is generally similar to the full range of unaltered Phanerozoic and Archaean shale^11^,^12^,^13^,^14^,^16^,^22^,^37^,^38^,^39^, which might be expected if the data reflect a transitional interval from relatively low to higher oxygen levels. Indeed, the data show an overall decrease in *δ*^82/76^Se with time, from the highest values in pre-Sturtian sediments, through to the lowest values at the Precambrian–Cambrian boundary (∼550 Myr ago). In detail, the data become isotopically lighter in the run-up to each of the three glaciations, but after the Marinoan glaciation, *δ*^82/76^Se is reset at higher values. This return to higher *δ*^82/76^Se is not apparent after the Gaskiers glaciation, but an excursion to moderately heavier values occurs during an euxinic interval in the early Cambrian Yangtze Basin. Where samples from two different basins overlap in age (that is, pre-Sturtian, immediately post-Sturtian and between the Marinoan and Gaskiers at 625–584 Myr ago, Fig. 5), we find a remarkable consistency of *δ*^82/76^Se values, which may suggest that our data represent global processes rather than local redox changes or diagenetic alteration. It has been proposed that Se isotopes can become highly fractionated during modern oxidative weathering of exposed, very Se-rich (≤26 wt%), outcrops^38^, where it appears that Se was oxidized and then re-reduced during aqueous transport. However, other studies of Se poorer soils have reported much less fractionation (<0.5‰)^40^, and the studies of large global data sets also suggest that isotopic effects of late-stage weathering of outcrops are probably not significant^12^,^16^. The highly fractionated values from the Se-rich soil^38^ may thus not be representative of all exposed surface environments. In any case, we present Se data from a combination of cores and outcrops^5^,^31^,^32^, and from different global localities, the combination of which should guard against significant late-stage diagenetic and weathering effects. In addition, for outcrop material, all weathered edges were cut away, and these samples often contain significant pyrite and Fe carbonate minerals suggesting minimal oxidation^5^, as confirmed by conventional and cathodoluminescence petrographic techniques^41^. Iron speciation data suggest that most of this study's samples were ferruginous, albeit with some euxinic conditions at the Precambrian–Cambrian boundary (Table 1)^5^,^42^.

## Discussion

The Se isotopic composition of volcanically derived Se is likely to be similar to that of the mantle (*δ*^82/76^Se ∼0–0.25‰ (refs 16, 43)). During oxidative weathering, relatively small fractionations of ∼0–0.4‰ occur^40^. However, during dissimilatory or abiotic oxyanion reduction in some modern and ancient lakes and rivers, isotopically light Se is sequestered in sediments, driving surface waters heavy by over 3‰ (refs 16, 44). Overall, modern ocean inputs are probably close to ∼0‰ on average, but may have differed in the past. Significant positive values in marine sediments from most of the earlier Precambrian may indicate that fluvial Se was isotopically heavy due to partial reduction during transport^16^. Marine plankton are thought to assimilate Se without fractionation and the isotopic composition of +0.42±0.22‰ in modern phytoplankton^12^ suggests that pre-industrial seawater SeO_*x*_^ 2−^ may have had *δ*^82/76^Se ∼+0.4‰ (refs 12, 16, 22). This elevated isotope ratio compared with the likely input must be balanced by accumulation of selenium with lower *δ*^82/76^Se in average marine sediments^12^.

Selenium can be preserved in sediments in three different forms. On a global scale, most modern selenium is biologically reduced by assimilation and deposited as Se_org_, with little inherent isotope fractionation (<0.9‰)^15^,^16^,^44^,^45^. Another component of sedimentary Se may be Se^IV^ adsorbed onto Fe-oxyhydroxides. This process also imparts a relatively small isotope fractionation of on average ∼0.15‰ (refs 45, 46). The third component comprises inorganic reduced phases (Se^0^ and Se^−II^ in sulphide minerals) that form via microbial dissimilatory reduction under suboxic conditions. Given that a significant fraction of the sedimentary Se is associated with organic matter^47^,^48^, any potential inorganic Se^−II^ or Se^0^ phases that formed by oxyanion reduction and may carry more negative *δ*^82/76^Se values become diluted^16^. This may explain why natural bulk sediments generally show much smaller fractionations relative to Se sources to the ocean than what is observed in laboratory experiments^16^.

**Figure 6:**
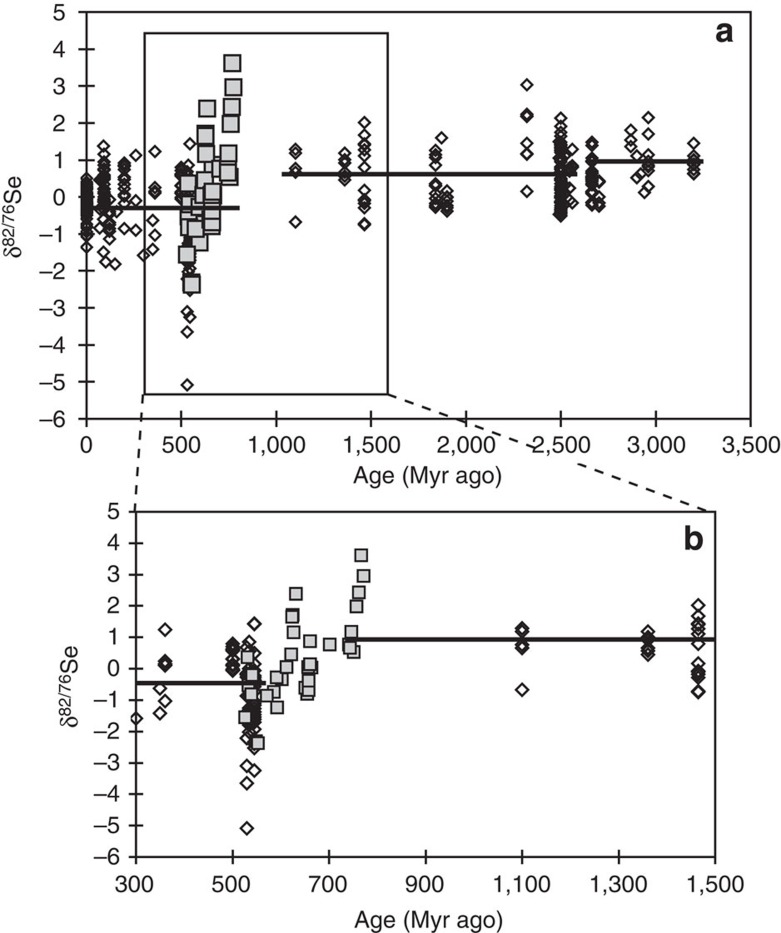
Compilation of all published global sedimentary Se isotope data from marine settings. (**a**) Published data (open symbols)^16^ compared with data from this study (closed squares) (*n*=463). The horizontal lines represent the mean from each time period^16^, demonstrating the overall trend towards isotopically lighter sediments after each major oceanic oxidation stage. (**b**) A focus of the same data compilation to the time period of this study. Here the black lines show the data mean from 1,500 Myr ago to the Sturtian (+0.92±0.76‰), and from the Gaskiers to the present (−0.32±0.85‰).

Isotopic fractionation of selenium species in sediments is related to the redox state of the overlying water. Both experimental^11^ and *ab initio* calculations^49^ have reported fractionation of Δ^82/76^Se_oxyanions−SeO_ ≤−21‰, although natural fractionation is considerably smaller^12^,^16^. In any case, *ab initio* equilibrium calculations likely do not reflect the largely kinetic fractionation likely occurring in natural systems^47^,^50^. The additional reduction of Se^0^ to selenide is not associated with significant isotope fractionation^11^. Thus, where dissimilatory reduction of SeO_*x*_^ 2−^ occurs, it sequesters lighter isotopes into sediments. If the site of the reduction (for example, suboxic sediments or oxygen-minimum zones (OMZs) in the water column) is linked to a large Se oxyanion reservoir, as, for example, the modern oxic ocean, then SeO_*x*_^ 2−^ reduction may be non-quantitative, such that sedimentary reduced phases preserve large negative fractionations^14^,^15^,^39^. Therefore, one would expect that an increase in the size of the dissolved SeO_*x*_^ 2−^ reservoir correlates with larger and more negative fractionations in locally suboxic environments. Selenium isotope compilations from across geological time statistically support this relationship, with mean isotope ratios heaviest in the mid-Archaean before the onset of oxidative weathering around ∼2.7 Gyr ago (*δ*^82/76^Se=+0.93±0.41‰, 1 s.d.; Fig. 6a), intermediate in the early to middle Proterozoic (+0.68±0.83‰, up to ∼1.1 Gyr ago), and lightest in the Phanerozoic (−0.30±0.89‰)^16^. Shorter-term trends in sedimentary Se isotope ratios confirming the same patterns have been reported from the latest Archaean^13^, the Permo–Triassic (P-Tr) boundary^39^, Mesozoic Oceanic Anoxic Events (OAEs; although these appear to exhibit basinal gradations^12^,^16^) and glacial–interglacial redox variations in the Cariaco Basin^22^.

The interpretation of a link between redox trends and Se isotopic composition may be complicated by temporal variations in the compositions of inputs to the oceans (dominantly riverine fluxes), and by local redox changes in restricted basins or marginal seas that are not representative of the global ocean. The former implies that seawater *δ*^82/76^Se has changed at key intervals in Earth history, as also suggested for Mo isotopes^51^,^52^. The latter is particularly important for Se due to its short marine residence time and its high sensitivity to changes in biological productivity^39^. Locally enhanced productivity may increase the relative proportion of Se_org_, which would lead to a smaller net fractionation of bulk sediments relative to seawater, because the dissolved SeO_*x*_^ 2−^ reservoir becomes locally depleted and because Se_org_ dilutes any fractionation carried by inorganic reduced phases. Hence, global, rather than local, redox conditions can only be inferred with confidence if similar trends and values occur in multiple different settings of similar age. Therefore, as with all tracers of marine redox conditions (for example, Mo isotopes^53^), Se isotopes provide information on local water column and water-sediment interface conditions, with an additional control from global seawater isotope ratios.

Published modern (<500 kyr old) data clearly show that sediments deposited in the oxic open ocean, or under well-connected OMZs, have isotopically lighter Se than those deposited in anoxic, restricted basin conditions^16^,^22^. The lighter values probably reflect the large SeO_*x*_^ 2−^ reservoir in oxic seawater, which allows for incomplete dissimilatory reduction under locally suboxic conditions, such as during early diagenesis in sediments or in OMZs. In contrast, sediments deposited under anoxic or euxinic conditions incur little to no isotopic fractionation, likely due to a combination of more quantitative SeO_*x*_^ 2−^ reduction, a higher proportion of Se_org_, and a local increase in the isotopic composition of dissolved SeO_*x*_^ 2−^ due to Rayleigh distillation^16^. In ferruginous systems, preferential removal of isotopically light Se by abiotic reduction with Fe(II) may play a role, with experimental results suggesting large fractionations, although it is unknown whether these experiments reflect the degree of fractionation in a natural state^11^,^14^,^16^,^46^,^54^. It has been suggested that light Se isotope ratios reported from the early Cambrian Yangtze platform may be due to this process^14^, but in this case the ferruginous reduction site was likely linked to a large Se oxyanion reservoir such as the global ocean or a deep oxic surface layer^16^,^46^. Overall, over geologic time, marine sediments have become isotopically lighter, in response to the overall oxidation of the ocean-atmosphere system (Fig. 6)^16^. The trend towards more negative values in our Neoproterozoic *δ*^82/76^Se data (Fig. 5) is thus most plausibly interpreted as deep-water oxygenation and an increase in the marine SeO_*x*_^ 2−^ reservoir towards the Precambrian–Cambrian boundary. Excursions from other time periods (latest Archaean^13^, P–Tr boundary^39^, OAEs^12^,^16^ and glacial–interglacial redox variations^22^) have been interpreted in a similar manner, lending support to our Neoproterozoic interpretation.

One key observation is that *δ*^82/76^Se values before the Sturtian and immediately after the Marinoan are isotopically heavier than both the mantle and average Precambrian values (Fig. 5). The highest Precambrian *δ*^82/76^Se value published before this study is +3.04‰ from ∼2.3 Gyr ago^16^, compared with our highest values of +3.63‰ at 0.765 Gyr ago, and positive values are generally observed in the Archaean^16^. Highly positive values are also reported from the first ‘whiff of oxygen'^13^ and during a local euxinic event shortly before the Permo–Triassic mass extinction^39^.

Several possibilities can be proposed for driving sediment *δ*^82/76^Se higher than crust or mantle values in the Precambrian. The first is that Precambrian rivers became isotopically heavy. Early rivers could have contained a large fraction of organically complexed Se, due to land-based microbial life. In this case, increasing atmospheric oxygen could have increased the SeO_*x*_^ 2−^/Se_org_ ratio, generating more reduction of SeO_*x*_^ 2−^ in rivers, driving the residue to higher *δ*^82/76^Se values^16^. This scenario is reasonable given that late Archaean lake and river sediments are isotopically lighter than marine sediments from the same time, indicating heavier continental surface waters^16^, assuming a similar fractionation mechanism in fresh and seawater. This phenomenon has also been reported from modern lakes with changing redox^44^. As during the late Archaean ‘whiff of oxygen'^13^, the pre-Sturtian *δ*^82/76^Se peak in our data set may thus reflect an atmospheric rise in pO_2_ above background levels without significant seawater oxygenation. A second possibility is that a Se isotope gradient with ocean depth could have formed, where continuous removal of light Se into sediments in shallower oxic waters would enrich offshore waters in heavy isotopes, also driving sediments downslope to isotopically heavier compositions. Such behaviour has been proposed to explain Archaean Se data^13^, as well as suggested for molybdenum isotopes^13^,^53^. However, it is only likely to apply here if the intracratonic basin represented by the Uinta Mountain and Chuar data had a universally deep chemocline everywhere in the basin. Hence, the isotopically heavy data might indicate that rivers became isotopically heavier at these times, likely due to relatively sudden increases in atmospheric O_2_ during the run-up to oceanic oxygenation. In fact, the most positive values around 770 Myr ago approximately coincide with a large perturbation in Cr isotopes, which has been interpreted as evidence of atmospheric oxygenation before the Sturtian glaciation^7^. Given that during the Marinoan CO_2_ levels appear to have increased^55^,^56^,^57^, and therefore O_2_ levels decreased, there could have been another O_2_ rise soon after the Marinoan, which may again have led to isotopically heavy Se in river waters. The increasing availability of oxygen is also consistent with high proportions of organic carbon burial in interglacial times^58^. Exceptionally high *δ*^82/76^Se is not observed after the Sturtian, but this may be because our samples are slightly older relative to deglaciation, compared with the post-Marinoan samples. A third possibility for high *δ*^82/76^Se values is high organic carbon burial. Enhanced biological productivity along continental margins could also have directly contributed to the observed heavy *δ*^82/76^Se after the Marinoan glaciations, because such conditions may have locally enhanced the proportion of Se_org_ in sediments.

**Figure 7:**
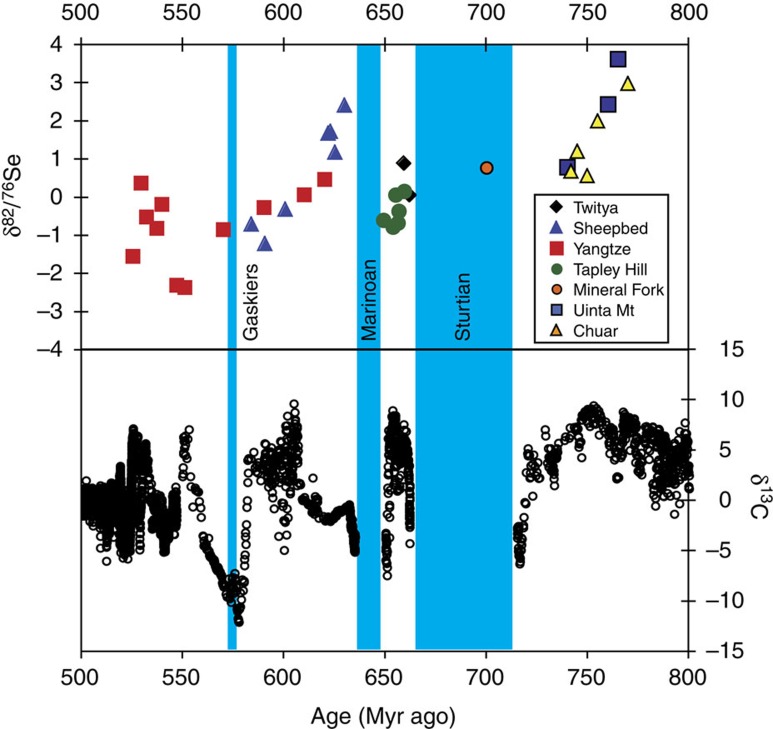
Comparison of this study's Se isotope data with composite C isotope data. Se isotopes suggest that the oxidation of the oceans took at least 100 Myr ago. The lack of correlation with *δ*^13^C suggests that the processes that control carbon (burial and oxidation of organic matter and carbonate dissolution/formation)^68^ appear not to be controlling Se isotopes, meaning that Se is not controlled by organic matter. The timing of the Sturtian has been altered according to ref. 4 (Sturtian is not shown as diachronous, as opposed to Fig. 5, for simplicity's sake).

Changing riverine Se isotope ratios are unlikely to have caused the trend towards isotopically light values observed in our samples, because there is no plausible mechanism that would drive river waters isotopically light. The few riverine sediments from the geologic past that have been measured have significantly lighter *δ*^82/76^Se than the crustal value^16^, implying isotopically heavy waters. Hence the negative fractionations in marine sediments are likely the result of *in situ* SeO_*x*_^ 2−^ reduction, either within sedimentary pore waters or in suboxic bottom waters. Importantly, the preservation of light *δ*^82/76^Se values relative to the crust implies that reduction was not quantitative. One possible explanation is a massive drop in productivity, such as observed during the P–Tr extinction^39^, which would lead to a decline in Se_org_ deposition. Although carbon burial may indeed have declined after its initial post-glacial boost^58^, there is no overall correlation between Se and C isotopes (Fig. 7). Therefore, the trend towards lighter values is probably a reflection of an increasing SeO_*x*_^ 2−^ reservoir in the Neoproterozoic ocean, that is, an increase in Se supply rather than a decrease in demand. The fact that multiple basins of similar age show similar patterns suggests that dissolved SeO_*x*_^ 2−^ concentrations increased globally, which is consistent with widespread oxygenation of the deep ocean.

A key complication in interpreting sedimentary Se isotopes (or indeed any redox proxy) is the influence of local redox changes in the water column, relative to global oceanic oxidation changes. Localized changes in redox (for example, ‘euxinic wedges') are thought to have occurred towards the end of the Cryogenian, especially at intermediate depths^5^,^59^,^60^. Such local changes would be assumed to complement or override Se isotope variations caused by global ocean changes, as, for example, also shown for redox tracers such as Mo and Cr isotopes^7^,^8^,^53^. In addition, the closer-to-quantitative capture of Se in more reduced settings would likely decrease the Se residence time before oxygenation^12^,^16^, enhancing local effects. The clearest method for attempting to determine a local versus global Se isotope signal is to compare sample suites from different global locations. Thus, similar *δ*^82/76^Se values between the data from Chuar and Uinta Mountain groups between ∼770 and 742 Myr ago suggest that, at least in the intracratonic basin associated with these groups, Se isotope ratios rapidly decreased. Immediately after the Sturtian (662–659 Myr ago), our Twitya and Tapley Hill samples give similar *δ*^82/76^Se values, and at times between the Marinoan and Gaskiers (∼625–584), similar isotope ratios are seen from the Sheepbed and Yangtze sample suites. Given that these suites are from different continents and different ocean depths, this suggests that Se isotope ratios between the Sturtian and Gaskiers generally represent global ocean phenomena (except, potentially, high *δ*^82/76^Se in the Sheepbed samples immediately after the Marinoan). Combined, these samples nevertheless suggest that global ocean sediments were becoming isotopically lighter during the course of the end-Cryogenian and Ediacaran.

Data after the Gaskiers glaciation only exist from the Yangtze Platform, and, therefore, there is nothing to prove that trends recorded here are more than local redox changes. However, the large *δ*^82/76^Se variation recorded from the Yangtze (from −2.3‰ to +0.4‰, followed by a return to negative values) provide a natural experiment that supports our interpretation of a redox control on *δ*^82/76^Se. At ∼540 Myr ago, Fe speciation suggests a localized, transient, ∼10 Myr interval of euxinia^5^. Once euxinia starts, *δ*^82/76^Se values increase substantially, and approach ∼0‰, reflecting near-quantitative removal of Se from seawater under euxinic conditions, in the form of sulphides and Se_org_ (refs 12, 13, 61). Sections from elsewhere in the Yangtze Basin display similarly high *δ*^82/76^Se values^14^ (Fig. 5). Then, once Fe speciation indicates a cessation of euxinia, *δ*^82/76^Se decreases again. Thus, sediments deposited under more reducing conditions have significantly higher *δ*^82/76^Se values than those deposited under more oxic conditions, as also implied by rapid^12^,^13^,^39^ and long-term^16^ oceanic trends.

Selenium's redox transitions result in Se isotopes being sensitive to redox conditions between those of S^−II^–S^IV^ and Fe^II^–Fe^III^. Hence, our data imply that at ∼770 Myr ago, coastal to intermediate depth (the depositional environment of the Chuar and Uinta Mt. groups) Eh was below the Se^IV^–Se^0^ transition, with reducing conditions widespread on shallow shelves, coupled to likely changes in the riverine isotope ratio, in response to atmospheric oxygenation. Afterwards, temporal trends in *δ*^82/76^Se imply that the oceans became progressively oxidized, as evidenced by deep marine depositional environments from different global locations showing increasingly lower *δ*^82/76^Se values. Global seawater reached a minimum around the Gaskiers glaciation (as shown by low values in both the Sheepbed and Yangtze), and potentially a lower minimum at the Precambrian–Cambrian boundary, although local effects cannot be distinguished here, because only one location was sampled from this time period. It is interesting to note that other samples of roughly similar age (also from the Yangtze Platform^14^) show even lighter Se isotope ratios (Fig. 6).

Overall, the Neoproterozoic data from this study fit well into the trends of Se isotope data over geologic time^16^ (Fig. 6). Samples before the Sturtian and after the Marinoan glaciations are isotopically heavier than the next youngest samples from ∼400 to 700 Myr ago earlier^16^. As discussed above, heavy values before the Sturtian could be a function of local conditions during intracratonic basinal deposition, where riverine influence on shallow waters would be relatively greater, and may not reflect wider global conditions. Heavy values after the Marinoan are also only reflected by one sample suite, but could also reflect changing continental weathering conditions during recovery from the ‘snowball' glaciation.

Hence, our data appear to capture an important transitional interval from high *δ*^82/76^Se values typical of the Archaean and early Proterozoic^12^,^13^,^16^ to the lighter values that characterise the Phanerozoic^12^,^14^,^16^,^22^,^35^,^38^,^39^ (Figs 5 and 6). This shift is consistent with oxygenation of the deep ocean during this time period, and in particular, suggests that the shift between fully ferruginous and fully oxic deep oceans took considerable time. The possibility that the trend towards lower *δ*^82/76^Se was caused by a relaxation in organic burial (and hence less heavy Se_org_ burial) is unlikely because there is no correlation between *δ*^82/76^Se and *δ*^13^C (Fig. 7).

The selenium isotope data imply that oxygenation was a protracted process. Iron speciation indicates that fully oxic deep waters only formed after the Gaskiers glaciation^5^. The Se isotope data suggest that this oxidation was spread over time, likely starting after the Marinoan, and taking around 100 Myr to pass through ferruginous and intermediate redox conditions (for example, manganous) to reach full deep-ocean oxia. Selenium's low abundance, multiple redox states and high redox potential may make Se isotopes a more sensitive redox proxy than tracers such as sulphur or molybdenum isotopes^16^, or ‘on-off' tracers such as Fe speciation. Hence, these data provide insight into the small degrees of redox change that led, perhaps in fits and starts, to full oceanic oxygenation. A Cryogenian–Ediacaran increase in oxygenation agrees with other redox-sensitive elements^10^,^62^,^63^ and biomarker evidence^64^. Thus, the significance of the Se isotope record is not only that it adds to growing evidence that the late Proterozoic and Cambrian ocean and atmosphere reached a progressively more oxic state, coinciding with the diversification of animal life, but also that the process of oxidation was protracted, and not ultimately triggered by the Gaskiers deglaciation, as other data suggest^5^.

In summary, this study has determined Se isotope ratios from shales deposited across the Neoproterozoic Oxygenation Event, from 770 to 525 Myr ago. Selenium isotopes are sensitive to changing oceanic redox conditions, both global and local, where lighter isotope ratios in sediments imply deposition from a water body that was linked to a large, oxic SeO_*x*_^ 2−^ reservoir. This hypothesis is supported by studies of other geologic time periods, as well as by local redox variations recorded by our post-Gaskiers samples. Overall, we find a trend of decreasing sediment *δ*^82/76^Se with time, and where time periods are covered by data from more than one sample suite, it suggests that the sediment Se isotopes reflect global redox changes. Hence, the data suggest that the global ocean became more oxic from at least the end of the Marinoan glaciation, and that, rather than a sudden redox change, the shift towards deep-ocean oxia was spread over a timescale of ∼100 Myr.

## Methods

### Analytical methods

The analytical methods used here for Se isotopes have been reported elsewhere^35^,^36^, but, briefly, two separate methods were used, where both report relative to NIST SRM-3149 (ref. 34). Samples younger than the Sturtian glaciation (∼670 Myr ago) were analysed by a double-spike inversion method, where international rock standard results were double-checked using different spikes and inversions, as well as doping and inverse modelling experiments^35^. The chemistry and analyses were performed at the Bristol Isotope Group, UK, using a Thermo Neptune MC-ICP-MS. Data from before the Sturtian were obtained using a sample-standard bracketing technique, with analyses performed on a Nu Instruments MC-ICP-MS at the University of Washington, USA^36^. To compare the techniques, the USGS shale standard SGR-1 was analysed by both methods. The former method yields *δ*^82/76^Se=+0.25±0.17‰ (*n*=16), and the latter +0.05±0.18‰ (*n*=9), meaning that values are identical within analytical uncertainty^35^,^36^.

## Additional information

**How to cite this article:** Pogge von Strandmann, P. A. E. *et al.* Selenium isotope evidence for progressive oxidation of the Neoproterozoic biosphere. *Nat. Commun.* 6:10157 doi: 10.1038/ncomms10157 (2015).

## Figures and Tables

**Figure 1:**
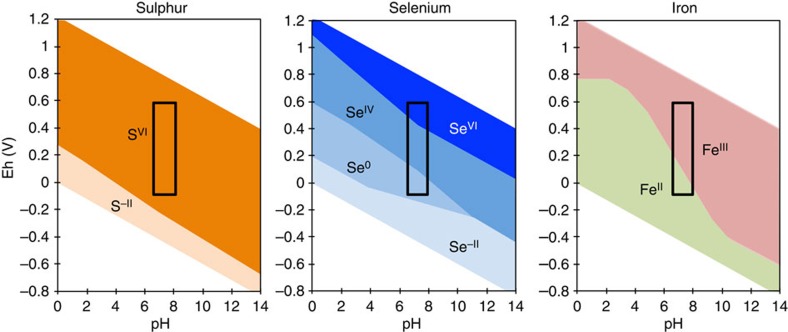
Redox speciation diagrams. Redox speciation of sulphur, selenium and iron. The black boxes show the range of Eh our Se isotope data suggest oceanic redox must have passed through during the interval 770–525 Myr ago ago^65^. The pH range also represents the oceanic conditions envisaged for this time interval^66^.

**Figure 2:**
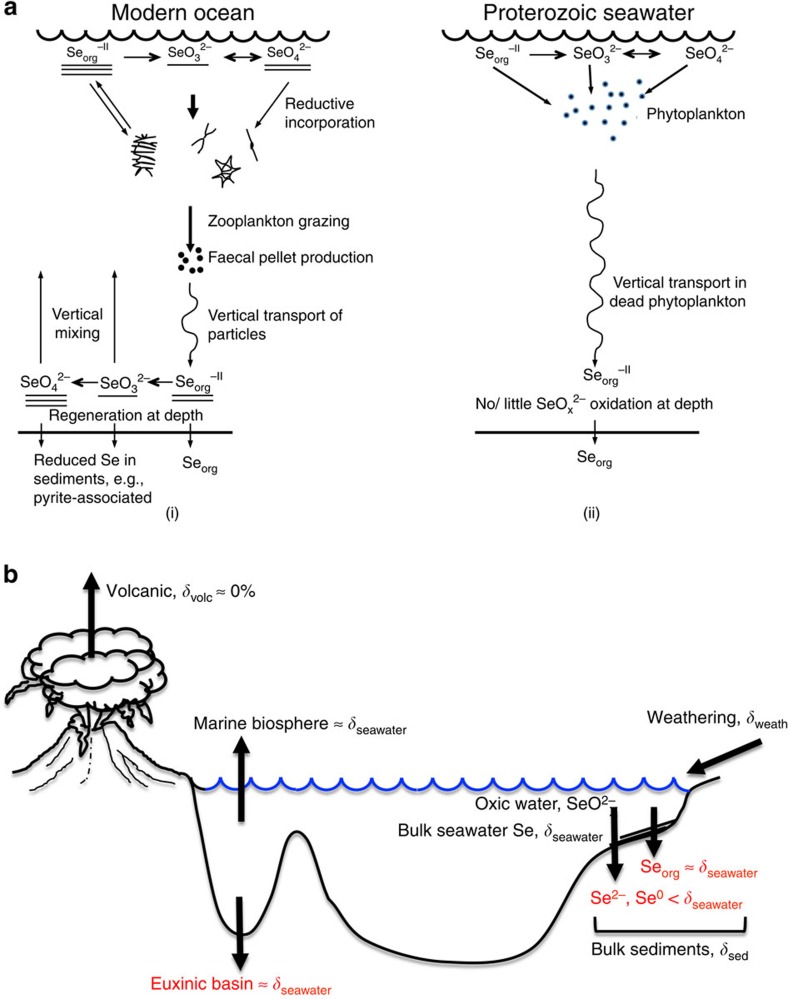
Summary of selenium and selenium isotope cycling in seawater. (**a**) (i) Modern cycling of Se in seawater, following Cutter and Bruland^47^. Underlines indicate relative concentrations in surface and deep water. Reduction of Se and incorporation into phases in sediments can impart negative isotope fractionation. (ii) Possible cycling of Se in Proterozoic seawater when the deep ocean was not oxygenated and had little SeO_*x*_^ 2−^. Reduction of Se from oxyanions would have been very limited. (**b**) A simplified diagram showing the main inputs and outputs in the geological selenium cycle^16^ (analogous to a very similar global scheme developed for isotopes of Mo, which is also a chalcophile element^67^). The main inputs to the ocean are volcanic and weathering fluxes with *δ*^82/76^Se values of *δ*_volc_ and *δ*_weath_, respectively^22^. A key output (highlighted in red) is the largely quantitative removal in isolated euxinic basins, which captures the bulk *δ*^82/76^Se value in seawater, *δ*_seawater_. Another key output, which we sample in our measurements, is removal of Se into sediments on slope or shelf environments in two key components that are mixed in the sediments with Se isotopic composition, *δ*_sed_^11^,^12^,^15^,^22^,^49^. The first form is organically bound selenium (Se_org_), which likely captures seawater isotopic composition, *δ*_seawater_. The second form is selenide (Se^2−^) or elemental selenium (Se^0^) produced from the microbial reduction of seawater selenite or selenate (SeO_*x*_^ 2−^). Because of isotopic fractionation, this second component tends to sequester lighter selenium isotopes. In the above diagram, we ignore small oceanic Se inputs from deep-sea hydrothermal sources and their outputs in the deep sea, because this Se does not mix into the oceans^21^. Cycling of Se from ocean to atmosphere and back via volatile biogenic organic selenides also does not cause isotope fractionation.

**Table 1:** Se concentration and isotope data for the analysed samples.

**Sample**	**Formation**	**Height,** **m**	**Age,** **Myr ago**	**TOC,** **%**	***δ***^**34**^**S**	**Se,** **μg g**^**−1**^	***δ***^**82/76**^**Se**	**2 s.d.**	**Redox conditions(based on Fe speciation)**
NO1-T1	Twitya	0	662.0	0.53	35.98	0.17	0.06	0.36	*f*
*rpt*						0.16	0.14	0.29	
NO1-T2	Twitya	60	659.3	1.18	26.33	0.53	0.89	0.38	*f*
NO1-T9	Twitya	330	639.1	0.18	20.46	0.02			*o-f*
NO1-T13	Twitya	460	630.1	0.10	29.67	0.03			*o-f*
NO1-T16	Twitya	550	623.8	0.18	22.33	0.05			*o-f*
NO1-T20	Twitya	700	613.4	0.15	27.75	0.08			*o-f*
N97-34-1	Sheepbed	5	630.0	0.64	−5.02	0.50	2.41	0.14	*f*
N97-34-10	Sheepbed	43	625.3	0.72	7.40	0.21	1.18	0.24	*f*
*rpt*						0.22	1.11	0.17	
N97-34-11	Sheepbed	60	623.2	0.67	16.76	0.15	1.73	0.25	*f*
*rpt*						0.16	1.65	0.21	
N97-34-12	Sheepbed	70	621.9	0.87	8.89	0.32	1.67	0.21	*f*
N97-34-15	Sheepbed	146	612.5	0.38		0.14			*f*
N97-34-17	Sheepbed	240	600.8	0.37	32.67	0.23	−0.31	0.22	*f*
N97-34-7	Sheepbed	322	590.6	0.47	7.84	0.28	−1.22	0.29	*f*
N97-34-20	Sheepbed	375	584.0	0.56	46.60	0.27	−0.71	0.22	*f*
N97-34-21	Sheepbed	450	574.7	0.32	33.86	0.03			*f*
N97-34-8	Sheepbed	468	572.5	0.39	30.59	0.14			*f*
Son 378	Niutitang (Xiaosi member)	117.15	524.8		16.17	0.04			*f*
Son 377	Niutitang (Xiaosi member)	116.25	525.2		12.20	0.13	−1.53	0.18	*f*
Son 367	Niutitang (Xiaosi member)	102.85	529.2	3.31	6.00	0.70	0.39	0.14	*f*
Son 358	Niutitang	74.12	532.0	8.03	15.90	4.9	−0.50	0.08	*f-e*
Son 389	Niutitang	52.15	537.0	6.82	0.20	9.3	−0.80	0.05	*e*
*rpt*						9.3	−0.89	0.07	
Son 384	Niutitang	48.5	539.5	9.64	7.37	39.5	−0.17	0.06	*e*
Son 326	Liuchapo	42.65	547.0	2.75	10.07	1.7	−2.29	0.08	*f*
Son 330	Liuchapo	37.05	551.0	13.51	0.90	15.3	−2.35	0.07	*f*
*rpt*						15.4	−2.24	0.09	*f*
Son 310	Doushantuo (Dengying)	26.2	570.0	5.48	0.00	5.5	−0.84	0.11	*f*
Son 311	Doushantuo (Dengying)	25.1	590.0	10.50	−1.32	6.5	−0.25	0.12	*f*
Son 312	Doushantuo	24.1	610.0	12.71	−8.60	6.2	0.08	0.11	*f*
Son 314	Doushantuo	22.2	620.0	4.41	−1.24	32.5	0.48	0.08	*f*
*rpt*						32.5	0.40	0.11	
Scywla 1280	Tapley Hill	1280.3	653.7	0.33		0.35	−0.77	0.21	*o-f*
Scywla 1,300	Tapley Hill	1,300	655.0	0.32		0.32	0.08	0.23	*f*
SR6 520.8	Tapley Hill	520.7	649.1	0.21		0.35	−0.59	0.23	*f*
SR17.2 805	Tapley Hill	805.4	656.2	0.38		0.60	−0.66	0.06	*f*
SR17.2 815	Tapley Hill	815.3	656.5	0.36		0.60	−0.35	0.16	*f*
SR17.2 905.5	Tapley Hill	905.5	659.2	0.90		0.27	0.17	0.24	*f*
SDH10*	Mineral Fork		700	0.53	5.57	0.32	0.79	0.26	*f*
16SL01*	Uinta Mt Gp, Deadhorse Fm		760	0.04	14.13	0.31	2.45	0.41	*f*
MH6-23-08-1*	Uinta Mt Gp, Moosehorn Lake Fm		765	0.16	6.49	0.13	3.63	0.00	*f*
RP01B12*	Uinta Mt Gp, Red Pine Fm		740	0.33	9.82	0.28	0.80	0.05	*f*
CC-16*	Chuar Gp, Awatubi Mbr		750	0.14	12.73	0.13	0.56	0.19	*f*
LCB-28*	Chuar Gp, Jupter Mbr		760	0.02	16.08	0.08	2.99	0.52	*f*
11-53-8*	Chuar Gp, Carbon Canyon Mbr		755	1.92	0.10	0.82	2.00	0.05	*f*
10-60-68*	Chuar Gp, Walcott Mbr		742	1.99		2.16	0.68	0.17	*f*
10-60-20*	Chuar Gp, Awatubi Mbr		745	0.69	17.00	0.29	1.20	0.26	*f*

e, euxinic; f, ferruginous; f–e, ferruginous–euxinic boundary; o–f, oxic–ferruginous boundary^60^; TOC, total organic carbon.

Additional information from refs 5, 31. All Se isotope data analysed by the double-spike method of Pogge von Strandmann *et al.*^35^, except those marked by *, where the method is that of Stüeken *et al.*^36^. The final column details the depositional redox conditions, based on Fe speciation^5^,^30^,^42^.
